# Reconstructing Defects of the Lower Lip: An Emphasis on the Estlander Flap

**Published:** 2016-12-24

**Authors:** Demetrius M. Coombs, Debra A. Bourne, Francesco M. Egro, Mario G. Solari

**Affiliations:** ^a^Drexel University College of Medicine, Philadelphia, Pa; ^b^Department of Plastic Surgery, University of Pittsburgh School of Medicine, Pittsburgh, Pa

**Keywords:** skin cancer, lower lip reconstruction, commissure involvement, Estlander flap, skin grafting

## DESCRIPTION

A 68-year-old man presented with squamous cell carcinoma of the right lower lip. Half of the right lower lip, one third of the right upper lip, the entire right oral commissure, and a large area of buccal surface were resected (1-cm margins). An Estlander flap was selected for reconstruction.

## QUESTIONS

1. **When performing reconstruction of lip defects, what considerations are of key importance?**
2. **A variety of reconstructive options exist for full-thickness defects of the lower lip. What guides the choice of reconstruction and what options (in general) are available?**
3. **Why was the Estlander flap selected to reconstruct this patient's lower lip?**
4. **What postoperative complications are most commonly associated with lower lip reconstruction?**

## DISCUSSION

Reconstructing lip defects requires a solid understanding not only of anatomy but also of function. The upper and lower lips work as a sphincter, facilitating mastication and phonation. The upper lip protects dentition, and the lower lip prevents the unwanted egress of oral secretions.^1^ Successful reconstruction strives to preserve oral competence, maximum oral aperture, labial mobility, and sensation, in addition to optimizing cosmesis.^2^ This allows patients to speak, eat, and retain expressive function.

Classification of lower lip defects is essential to selecting a reconstructive option. Key parameters include location, length, width, and depth. Depth refers only to vermillion and partial-thickness (superficial muscular extension) and full-thickness defects—full-thickness lesions are further described according to whether they involve less than one third (small), one third to two thirds (medium), or more than two thirds (large) of the lower lip. Another important consideration is whether the lesion is central or lateral or involves either commissure.^1-3^ The literature describes more than 100 lip reconstruction procedures,^3^ and the “reconstructive ladder” aids selection of an appropriate technique. In defects comprising less than one third of the lip, primary closure produces a functional and aesthetic outcome. Realignment of the vermillion border and reapproximation of the orbicularis oris muscle layers remain 2 crucial goals.^4^ Local flaps represent a well-suited option for defects affecting one third to two thirds of the lip.^1-4^ When the defect exceeds two thirds of the lip (subtotal), or involves the entire lip, regions with prior reconstruction, or irradiated tissue, free-flap reconstruction is recommended. Options include the radial or ulnar forearm and anterolateral thigh (ALT). To ensure lower lip suspension, simultaneous tendon transfer (palmaris longus or flexor carpi radialis) or thigh fascia may be required.^4^

**Figure 1:**
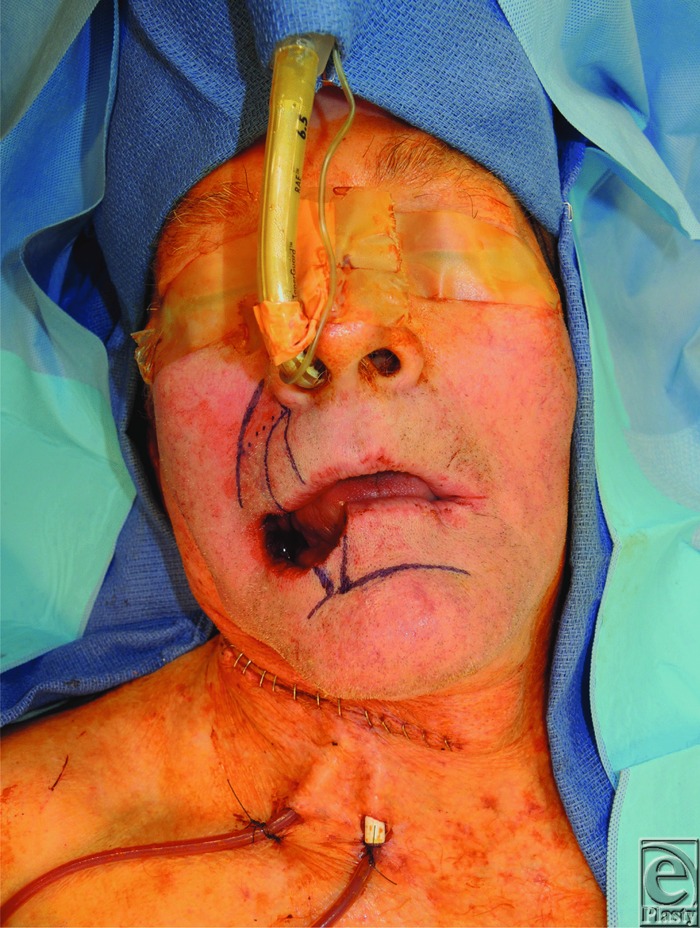
Design of the Estlander flap intraoperatively (immediately postresection by ENT).

This patient presented with a full-thickness, medium-sized defect and commissure involvement—both reasonable indications for the cross-lip Estlander flap (based on the labial artery and shown in Fig 1).^1,2^ An additional advantage of the Estlander flap is the avoidance of less desired scar patterns associated with more elaborate, bilateral local flaps.^4^ Ebrahimi et al^5^ demonstrated that reconstructing commissure defects with the Estlander flap yielded an 80% satisfaction rate and no reported functional defects (the patients complained only of lip asymmetry).

Postoperative complications following lower lip reconstruction vary according to procedure. In the case of the Estlander flap and other local flaps, the following remain important: flap loss, blunting of the repaired commissure, lip asymmetry, sensory loss, hypersensitivity, edema, microstomia, poor oral competence with drooling, and undesirable scarring.^2,5^ Yamauchi et al^6^ described a modification to the Estlander flap whereby lip and commissure symmetry was maintained postoperatively but recovery of sensory and contractile function was incomplete.

**Figure 2:**
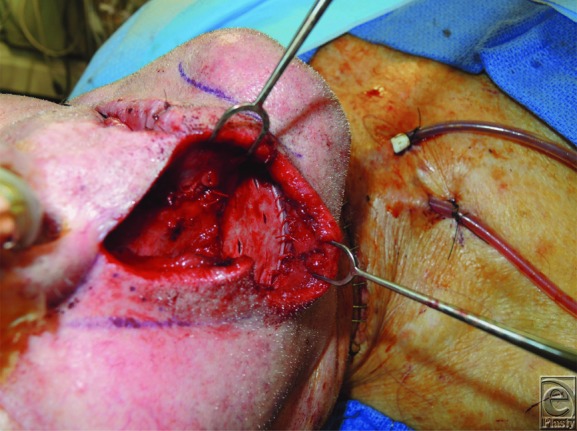
Split-thickness skin graft from anterolateral thigh inset to cover right buccal mucosal defect prior to final closure.

**Figure 3:**
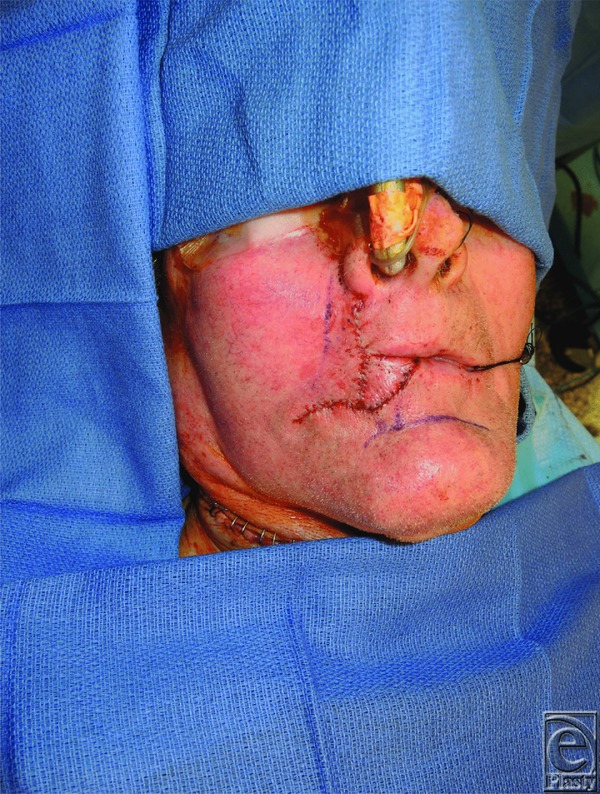
Immediately following Estlander flap inset and final skin closure.

**Figure 4:**
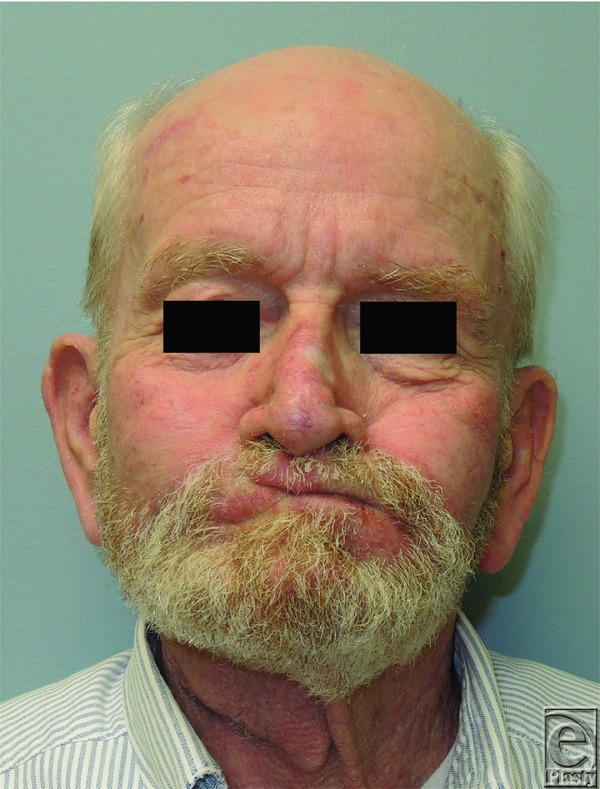
Approximately 2 months postoperatively (note that the patient is demonstrating complete lip seal while puffing his cheek).

Carcinomas of the lip represent 25% to 30% of oral cancers, involve the lower lip 90% of the time, and contain squamous cell carcinoma in 95% of cases—surgical resection is the standard of care.^3^ A successful reconstruction addresses cosmesis and function, ultimately preserving quality of life. The location, length, width, and depth of the lip defect, in conjunction with the “reconstructive ladder,” serve as a guide for selecting an appropriate reconstructive technique. Described in 1872, the Estlander flap was used in this patient primarily as a consequence of commissure involvement.^7^ Reconstruction of the buccal mucosal defect was also important to limit contracture—a split-thickness skin graft was harvested from the patient's thigh and inset prior to closure (Figs 2 and 3). At 2 months postoperatively, complete lip seal was maintained (Fig 4). Each reconstructive option poses unique complications, and regardless of the defect and requisite procedure, appropriate preoperative counseling remains imperative to surgical planning and managing patient expectations.
